# Impact of migalastat therapy on corneal deposits in a female with Fabry disease: A case report

**DOI:** 10.1016/j.ymgmr.2026.101318

**Published:** 2026-05-14

**Authors:** Nicola Vitturi, Giorgia Gugelmo, Francesca Zanini, Gianni Carraro, Livia Lenzini, Andrea Leonardi

**Affiliations:** a Division of Metabolic Diseases, Department of Medicine, University Hospital of Padua, Padua, Italy; b Department of Neurosciences, Ophthalmology Unit, University of Padua, Padua, Italy; c Division of Nephrology, Department of Medicine, University Hospital of Padua, Padua, Italy; d Department of Medicine, University of Padua, Padua, Italy

**Keywords:** Fabry disease, Cornea verticillata, Migalastat, In vivo corneal confocal microscopy

## Abstract

Fabry disease is a rare X-linked lysosomal storage disorder caused by alpha-galactosidase A deficiency, leading to globotriaosylceramide accumulation in multiple organs, including the eye, where corneal verticillata represents a typical sign. This case report examines the effect of migalastat on corneal deposits in a female who experienced therapy interruption during two pregnancies. It emphasizes the importance of multidisciplinary care with ophthalmologic follow-up and explores long-term effects of migalastat on corneal deposits and disease progression.

## 1 Introduction

Fabry Disease (FD) is an inherited metabolic disorder caused by mutations in the *GLA* gene, which encodes for the enzyme alpha-galactosidase A (*α-GAL A*). The deficiency of this enzyme leads to the accumulation of globotriaosylceramide (Gb3) in various organs, including the kidneys, heart, skin, and cornea. Cornea verticillata, or whorled corneal deposits, is a common early finding in FD and is often used as a diagnostic clue by slit lamp examination and by the use of in vivo corneal confocal microscopy (IVCM) [1,2]. IVCM, an imaging tool, has the ability to demonstrate the presence of corneal Gb3 deposits even in the absence of slit lamp finding and to show a small-fiber neuropathy in the cornea of FD patients [2]. Migalastat, a pharmacological chaperone, was introduced as a treatment for FD to stabilize the dysfunctional α-GAL A enzyme, reducing Gb3 accumulation in tissues [3]. This case report explores the management of Fabry disease in a 37-year-old female, focusing on the impact of migalastat on corneal deposits with interruptions in therapy during pregnancy.

## 2 Case presentation

A 37-year-old female was diagnosed with FD in 2004 after the detection of cornea verticillata. Genetic testing confirmed a heterozygous variant in the *GLA* (galactosidase alpha) gene (p.Met290Thr, c.869 T > C), a pathogenic missense mutation [4]. At the time of diagnosis, her *α-GAL A* activity was significantly reduced (2.91 μM/h), and elevated lyso-Gb3 levels (4.46 nmol/mL) were noted. Additionally, a skin biopsy confirmed the diagnosis of FD. The patient undergoes regular follow-up visits, including annual multidisciplinary evaluations involving specialists in internal medicine, nephrology, cardiology, dermatology, and ophthalmology.

The patient's clinical course remained relatively stable until the age of 31, when cardiac magnetic resonance imaging (MRI) showed reduced native T1 values in the myocardium without other signs of FD cardiomyopathy and an electrocardiogram revealed sinus bradycardia and a short PR interval.

**Fig. 1:**
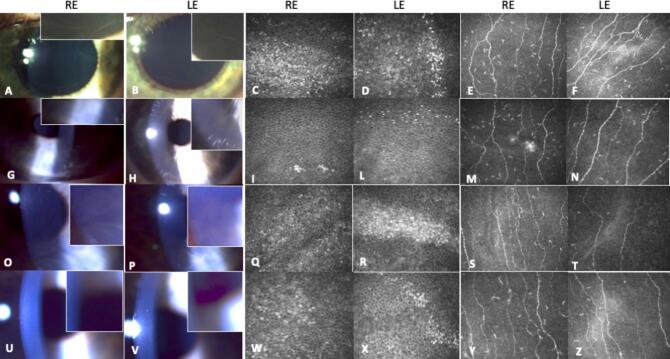
Cornea verticillata in the 34-year-old female before starting therapy with migalastat (A-F) and after one year of therapy (G-N). Corneal deposits by slit lamp examination in the right eye (RE) (A), and in left eye (LE) (B) with higher magnification of the corneal deposits in the boxes. In vivo corneal confocal microscopy (IVCM) demonstrate hyper-reflective intracellular deposits at the level of basal epithelium fulfilling the whole cytoplasm in the right and left eye respectively (C and D). E and F show abnormalities of the sub-basal nerve plexus in both eyes with increased nerve tortuosity. After a year of therapy, corneal deposits were significantly reduced by both slit lamp examination (G = RE; H = LE) and IVCM (I and M = RE; L and N = LE). Two years later after discontinuation of migalastat because of the second pregnancy, corneal deposits (O, P) and IVCM demonstrate, similar hyper-reflective intracellular deposits (Q, R) and sub-basal nerve plexus tortuosity (S, T) as before starting any therapy. After migalastat re-introduction, corneal deposits were reduced again as show by slit lamp (U, V) and IVCM (W-Z).

As cardiac manifestations began to emerge, treatment with oral therapy (migalastat, mutation-amenable) was indicated, but initiation was delayed due to the first pregnancy. Cornea verticillata was first identified at the time of diagnosis and confirmed on subsequent ophthalmological evaluations. At the time cardiac manifestations emerged and treatment was being considered, corneal deposits remained present (Fig. 1 A-F). At the age of 32 years she delivered a healthy female infant via caesarean section at 39 weeks of gestation. Therefore, migalastat therapy was introduced after the cessation of breastfeeding at the age of 34 years, with the patient initially taking one capsule = 123 mg every other day. After one year of therapy, ophthalmological assessments revealed significant improvement in corneal deposits, which had either diminished or were no longer visible (Fig. 1 G-N).

However, after nearly two years of treatment, migalastat therapy was discontinued with the onset of the second pregnancy, with the birth of another healthy child at the age of 35 years. 18 months later, at the most recent multidisciplinary follow-up visit (37-year-old), the patient was still breastfeeding and, as recommended, she remained off therapy. Ophthalmological examination and IVCM imaging confirmed that the corneal deposits had reformed but reduced in comparison to earlier evaluations prior the start of the treatment (Fig. 1 O-T). Visual acuity was preserved (OD 10/10, OS 10/10), intraocular pressure was 11 mmHg bilaterally and fundus examination was normal. When she stopped breastfeeding, migalastat therapy was restarted. Nine months later, a new ophthalmological evaluation, including slit-lamp photography and IVCM, documented once again an almost complete disappearance of corneal deposits on slit-lamp examination (Fig. 1 U-Z), with deposits still detectable but significantly reduced on IVCM. Additionally, lyso-Gb3 levels were reassessed and found to be stable.

## 3 Discussion

This case report describes a female patient whose initial diagnosis was based on the presence of cornea verticillata, a typical and sensitive ocular sign of FD [5]. This is consistent with previous findings that suggest its high diagnostic sensitivity [6,7]. Genetic testing confirmed a heterozygous mutation, p.Met290Thr in the *GLA* gene, which has been reported as pathogenic mutation [4].

Treatment options for FD have evolved over the years. Enzyme replacement therapy (ERT) and oral chaperone therapy are now standard approaches for mutation-amenable patients [3,8]. Migalastat is a pharmacological chaperone that stabilizes the mutant *α-GAL A* enzyme, allowing it to function more efficiently. In vitro and In vivo studies have demonstrated its efficacy in patients with specific mutations, including the pathogenic p.Met290Thr variant [9]. Migalastat treatment has shown improvements in biomarkers, organ function, and in some clinical manifestations [3,10].

Ophthalmological evaluation remains a remains key in monitoring FD. Corneal deposits, though rarely impairing vision, indicate systemic involvement and may reflect therapy effects [[11], [12]], especially when treatment is modified or discontinued. Regular eye examinations, including slit-lamp biomicroscopy and IVCM, are essential for detecting early changes in corneal deposits and assessing their evolution under different treatment regimens [2,5]. In this case, the patient's response to migalastat therapy was favourable, with significant improvement in corneal deposits observed within the first year of treatment. This is consistent with other studies demonstrating that migalastat can reduce renal and cardiac FD manifestations in mutation-amenable patients [[13], [14], [15], [16]]. Cessation of therapy during the second pregnancy led to a reformation of deposits, though less pronounced than before treatment, which disappeared again after migalastat was re-started. Although corneal deposits are not associated to systemic severity, this underlines the importance of careful monitoring and the ophthalmologist's role.

A multidisciplinary approach ensures comprehensive care across all affected organ systems and a dedicated team supports shared decisions on therapy management, with input from all relevant specialties [17].

In literature, migalastat treatment has not been shown to reverse corneal manifestation of FD; yet in our case, it was successful in reducing corneal deposits, appearing to clinically follow a therapy-suspension-re-initiation pattern despite metabolic and organ functions were stable.

In conclusion, this case highlights both the effectiveness of migalastat in reducing corneal deposits in a mutation-amenable patient and the dynamic and reversible nature of these ocular manifestations with treatment suspension and re-initiation.

## CRediT authorship contribution statement

**Nicola Vitturi:** Writing – original draft, Methodology, Data curation, Conceptualization. **Giorgia Gugelmo:** Writing – original draft, Investigation, Data curation. **Francesca Zanini:** Validation, Investigation. **Gianni Carraro:** Validation, Project administration, Conceptualization. **Livia Lenzini:** Writing – review & editing, Validation. **Andrea Leonardi:** Writing – original draft, Supervision, Methodology, Investigation, Data curation, Conceptualization.

## Declaration of competing interest

None.

## Data Availability

Data will be made available on request.
